# Silkworm (*Bombyx mori* L.) Has Beneficial Effects on Menopausal Symptoms by Enhancing Estrogen Receptor Signaling in Ovariectomized Mice

**DOI:** 10.3390/nu16132164

**Published:** 2024-07-08

**Authors:** Sung Jin Kim, Mi-Gi Lee, Joohwan Lee, Myoung-Sook Shin

**Affiliations:** 1 College of Korean Medicine, Gachon University, Seongnam 13120, Republic of Korea; sungjinkim001@gmail.com; 2 Bio-Center, Gyeonggi-do Business and Science Accelerator, Suwon 16229, Republic of Korea; 3 Healthy Spoon Research Institute, 160, Hyanggyo-ro, Paldal-gu, Suwon 16263, Republic of Korea

**Keywords:** *Bombyx mori* L., menopause, silkworm powder, estrogen receptor signaling

## Abstract

Existing hormone replacement therapy for menopause has drawbacks, necessitating new treatment agents. Silkworms have demonstrated estrogenic properties, offering promising alternatives. We assessed the therapeutic effects of freeze-dried silkworm powder (SWP) on menopausal symptoms using an ovariectomized (OVX) mouse model. The experimental design comprised a sham surgery group (Sham), an OVX control group, a low-dose SWP group post-OVX (80 mg/kg, OVX-SWP-L), a high-dose SWP group post-OVX (160 mg/kg, OVX-SWP-H), and an estradiol treatment group post-OVX (OVX-E2). Treatments were administered orally thrice weekly over eight weeks; body weight was monitored weekly. The SWP-treated groups (SWP-L and SWP-H) exhibited less weight gain and increased uterine thickness than the OVX control. Molecular analyses demonstrated that SWP significantly enhanced the phosphorylation of estrogen receptor alpha (ERα), ERK, and AKT. Furthermore, biochemical assays revealed reduced serum neutral lipids across all SWP treatment groups. Notably, HDL-cholesterol levels were significantly increased in the SWP-L group compared to the OVX group. Serum estradiol concentrations were elevated in all the SWP groups, with significant increases in the high-dose group. These findings indicate that SWP may promote the activation of estrogen receptor signaling and improve symptoms associated with estrogen deficiency during menopause.

## 1. Introduction

Menopause is the cessation of menstruation in women, which is recognized one year after the final menstrual cycle [1]. Perimenopause refers to the 3–4-year transitional period around menopause during which ovarian function progressively declines. During this phase, estrogen levels decrease below the required physiological threshold. Estrogen is pivotal in regulating body energy balance and metabolic processes, modulating cell proliferation, immune and osteoblastic cell activation, neural progenitor cell activation, and epithelial cell regeneration. Consequently, the hormonal changes that occur during perimenopause cause a spectrum of menopausal symptoms, including facial flushing, urinary disorders, osteoporosis, dyslipidemia, depression, cognitive decline, insomnia, and skin aging [2,3]. Furthermore, the decrease in estrogen is associated with increased accumulation of abdominal fat, leading to central obesity, which elevates the risk of type 2 diabetes, hypertension, hypertriglyceridemia, elevated low-density lipoprotein (LDL) cholesterol levels, and cardiovascular diseases [4,5].

Mulberry silkworms, the larvae of *Bombyx mori* L. from the Bombycidae family, contain abundant essential amino acids and unsaturated fatty acids, making them beneficial for improving liver health, enhancing blood circulation, and serving as functional food ingredients [6]. In addition to traditionally being utilized in Asia for skin beauty, liver function enhancement, immune boosting, and fatigue recovery, silkworms have also been employed as therapeutic agents for various ailments [7,8,9,10,11,12]. Furthermore, the historical literature corroborates their efficacy in treating hypertension and diabetes [13]. Studies have indicated that protein hydrolysates derived from silkworm pupae comprise bioactive peptides with antioxidant and anticancer properties, which potentially reduce cellular damage caused by reactive oxygen species and DNA alterations, contributing to alleviating diseases such as Alzheimer’s disease, Parkinson’s disease, essential hypertension, atherosclerosis, diabetes, and rheumatoid arthritis [14,15,16,17]. Notably, silkworm extracts have been demonstrated to effectively inhibit hydroxyl radicals and LDL cholesterol, reduce the arteriosclerosis index and lipid peroxidation, and enhance the activity of the defense enzyme superoxide dismutase (SOD), ultimately contributing to anti-aging effects [18,19]. The majority of middle-aged women typically experience menopausal disorders, which has prompted extensive research into various therapeutic and preventive strategies. Although hormone replacement therapy (HRT) is currently the gold standard of treatment, it is associated with increased risks of endometrial cancer, cardiovascular disease, and breast cancer due to elevated estrogen levels [20]. Studies by the National Institutes of Health (NIH) have further revealed that HRT may increase the risk of breast cancer, venous thromboembolism, coronary artery disease, and stroke [21,22]. Thus, there is an urgent need to develop natural or composite agents capable of mitigating menopausal symptoms and preventing chronic diseases, and current research is exploring these possibilities [23,24]. For instance, Quynh et al. reported the estrogenic activity of silkworm extracts obtained using 30% ethanol [25]. This study aimed to contribute to this line of investigation by assessing the effect of freeze-dried silkworm powder (SWP), administered orally three times a week for 8 weeks, on menopausal symptoms in ovariectomized (OVX) mice to evaluate various menopausal-related indicators.

## 2. Materials and Methods

### 2.1. Preparation of Silkworm Powder

Silkworm extract, obtained from 3–5-day-old instar larvae, was procured from a sericulture farmhouse from the Yecheon Sericulture Cooperative Association (May 2021, Yecheon, Gyeongsangbuk-do, Republic of Korea). The larvae were freeze-dried at −80 °C for 30 h and subsequently ground into powder using a pin mill grinder.

### 2.2. Evaluation of ABTS Radical Scavenging Activity

The measurement of antioxidant activity using ABTS radicals is based on the principle that antioxidants in the sample remove ABTS free radicals, resulting in the decolorization of the turquoise color (1). A solution containing 7 mM ABTS and 2.45 mM potassium persulfate was allowed to react in the dark for 16 h to generate radicals. SWP was diluted to concentrations of 15.6, 31.3, 62.5, 125, 250, 500, and 1000 µg/mL, and 10 µL of each concentration was added to 290 µL of ABTS solution in a 96-well microplate. After mixing, the reaction was allowed to proceed in the dark for 6 min, and absorbance was measured at 734 nm to calculate scavenging activity. The ABTS radical scavenging capacity of the samples was determined using the following equation.

$$\mathrm{ABTS}\;\mathrm{radical}\;\mathrm{scavenging}\;\mathrm{activity}\;(\%)=\frac{\left(Control\;O.D.\right)-(Sample\;O.D.)}{\left(Control\;O.D.\right)}$$

### 2.3. Experimental Design

**Figure 1:**
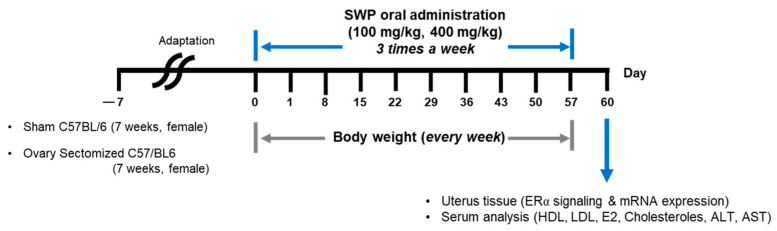
Experimental schedules for evaluation of SWP on menopausal symptoms.

Female 7-week-old C57BL/6 mice with both ovaries resected were purchased from SLC (Shizuoka, Japan) and supplied by JoongAng Animal Experiment Co., Ltd. (Seoul, Republic of Korea). A sham operation was performed on the non-oophorectomized control group. The mice were housed in a controlled environment (temperature: 22 ± 3 °C; relative humidity: 55 ± 5%; and a 12 h light/dark cycle). Additionally, the mice had ad libitum access to a commercial diet (Orientbio, Seongnam, Republic of Korea) and water. The animals were acclimatized for 7 days before experimentation. The experimental groups included sham surgery (Sham), ovariectomized control (OVX), low (80 mg/kg bw; OVX + SWP-L) and high (160 mg/kg bw; OVX + SWP-H) dosages of silkworm powder suspended in 0.5% Methyl Cellulose 400 solution (0.5% MC) (Wako, Tokyo, Japan), and a group that underwent ovariectomy and received estradiol (1 mg/kg bw; OVX + E2). Each group comprised five mice. The treatments were administered orally thrice weekly for eight weeks, and body weight was recorded weekly, as shown in Figure 1. All animal experiments were performed in accordance with the guidelines of the Institutional Animal Care and Use Committee of Gachon University (Approval No. GU1-2022-IA0031-00).

### 2.4. Uterine Tissue and Blood Collection

After the final administration, the animals were fasted for 12 h and anesthetized using ether. Approximately 500 μL of blood was collected, and the uterus was excised immediately. The blood samples were stored in BD Vacutainer SST Blood Collection tubes (Becton Drive, Franklin Lakes, NJ, USA) at room temperature for 30 min, centrifuged at room temperature at 400× *g* for 20 min to separate the serum, and subsequently stored at −80 °C. The uterus was weighed and photographed, and the thickness was measured at two sites.

### 2.5. Assay of Serum 17β-Estradiol

Estradiol content in serum was analyzed using the Mouse Estradiol ELISA Kit (Abcam, ab285237, Waltham, MA, USA), according to the manufacturer’s instructions. Briefly, the serum samples were mixed with 17β-estradiol buffer containing antigen-enzyme conjugates for 1 h, followed by incubation with a color solution for 2 min. The reaction was stopped using a stop solution, and absorbance was measured at 450 nm using a TECAN microplate reader (Morrisville, NC, USA).

### 2.6. Preparation of Tissue Lysate for Immunoblotting

Uterine tissues were stored at −80 °C. The tissues were thawed on ice, placed in a disposable biomasher (TAKARA, Tokyo, Japan), and homogenized in RIPA buffer containing protease inhibitors and 1 mM DTT. After centrifugation (13,000 rpm, 20 min, 4 °C), the supernatant was collected, and the protein concentration was determined. The samples were diluted to a concentration of 1 mg/mL in RIPA buffer, mixed with 4× Laemmli Sample Buffer (Bio-Rad, Hercules, CA, USA), denatured, and subjected to SDS-PAGE (Mini-Protean TGX, Precast Gel, Bio-Rad). The proteins were transferred to a PVDF membrane (Millipore, Burlington, MA, USA), and protein phosphorylation was detected using specific antibodies. Phosphorylation bands were quantified using image quantification software FUSION Solo S (Vilber Lourmat, Marne, France), graphed, and statistically analyzed. The antibodies used for the experiments are listed in Table 1.

### 2.7. Quantitative Real-Time Reverse Transcription Polymerase Chain Reaction (qRT-PCR)

The frozen uterine tissues were thawed on ice and homogenized in a disposable biomasher using RLT buffer, and the RNA was extracted using an RNA extraction kit (Qiagen, Germany). cDNA was synthesized using a RevertAid First Strand cDNA Synthesis kit (Thermo Fisher Scientific, Waltham, MA, USA). The mRNA expression level of estrogen receptor α (Esr1) was measured using a TaqMan gene expression assay (Mm00433149_m1), normalized to actin (Mm02619580_g1), and analyzed using a QuantStudio 3 real-time PCR system (Applied Biosystem, Waltham, MA, USA).

### 2.8. Blood Biochemical Analysis

Total cholesterol, triglyceride (TG), high-density lipoprotein (HDL), LDL cholesterol, and liver enzyme (ALT and AST) levels were outsourced to a specialized animal blood analysis company (OBEN, Suwon, Republic of Korea).

### 2.9. Statistical Analysis

All data are presented as the mean ± S.E. The statistical significance was evaluated using a two-way analysis of variance (ANOVA) for multiple comparisons, followed by Tukey’s test (*p* < 0.05 or *p* < 0.01 were considered statistically significant).

## 3. Results and Discussion

### 3.1. Preparation of Freeze-Dried SWP

The SWPs used in this study to prepare the powder were a mix of third-day larvae (males and females) from the fifth instar, cultivated by the Yecheon Sericulture Cooperative Association in May 2021 in the Republic of Korea. Immediately after cultivation, the silkworm pupae were freeze-dried at −80 °C for 30 h and ground into powder using a pin mill grinder for experimental use. Silkworms are a high protein source, containing pharmacologically significant amino acids such as serine, alanine, tyrosine, and aspartic acid [26]. Also, Eom et al. have already reported that that the physicochemical composition of silkworms comprises approximately 55–65% crude protein, 9–14% crude fat, 5.4% crude fiber, 9.3% ash, and 4.7% moisture, along with significant levels of minerals such as calcium, iron, and magnesium [27]. Thus, it can be suggested that the various nutritional components present in silkworm powder contribute to multiple physiological activities in vivo.

### 3.2. The Effect of SWP on ABTS+ Free Radical Scavenging Activity

**Figure 2:**
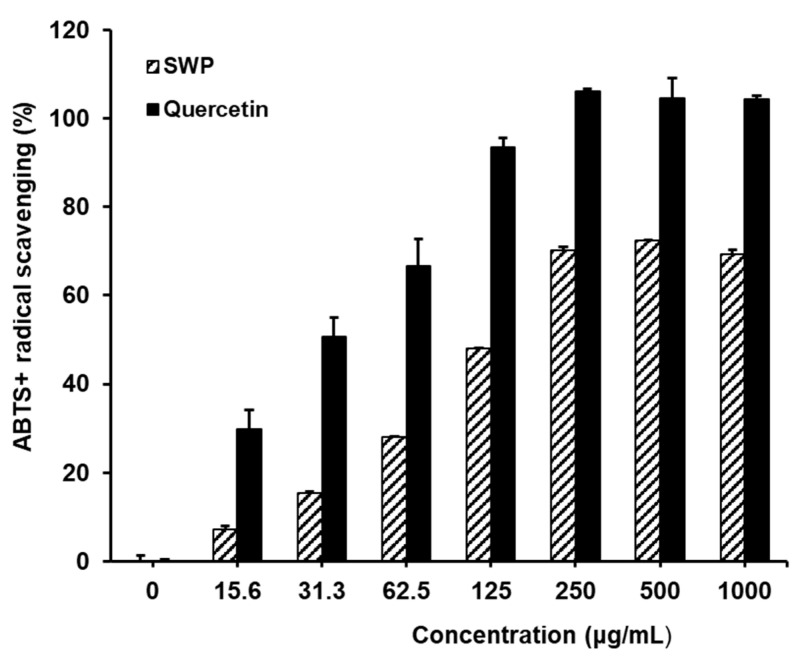
ABTS radical scavenging abilities of SWP. Results are mean ± SD of three independent experiments. Quercetin used for positive control of radical scavenging activity.

Assessment of the ABTS radical scavenging activity of SWP revealed that, in comparison to the control group, the positive control quercetin exhibited significant reductions in ABTS+ levels, with values of 29.8 ± 2.1, 50.6 ± 0.4, 66.5 ± 4.6, 93.5 ± 0.8, 106.2 ± 5.7, 104.5 ± 1.4, and 104.3 ± 8.6% at concentrations of 15.6, 31.3, 62.5, 125, 250, 500, and 1000 µg/mL, respectively (Figure 2). SWP treatment at these concentrations resulted in dose-dependent reductions in ABTS+ levels, with values of 7.3 ± 0.0, 15.5 ± 0.8, 28.0 ± 0.1, 48.0 ± 1.0, 70.1 ± 1.9, 72.3 ± 0.6, and 69.4 ± 1.7%. These findings demonstrate the efficacy of SWP in scavenging ABTS+ free radicals and highlight its potential as an antioxidant agent. Further investigation into the specific compounds responsible for the antioxidant activity of SWP, such as polyphenols and flavonoids, would provide valuable insights into the mechanisms underlying its free radical scavenging properties and guide future research on its potential therapeutic applications.

### 3.3. Change in the Body Weight of OVX Mice Following the Administration of Freeze-Dried Silkworm Powder

Postmenopausal women experience an increase in blood cholesterol and visceral fat due to the reduced cholesterol conversion to estrogen, leading to increased abdominal fat [28,29]. Similarly, ovariectomized laboratory animals (OVX model) tend to gain weight owing to a lack of estrogen. Particularly, the increase in abdominal fat contributes to a higher risk of metabolic syndrome and cardiovascular diseases [30]. In this study, both the sham-operated and OVX mice received the positive control (17β-estradiol) or freeze-dried SWP thrice weekly for 8 weeks, and the total body weight change over this period was monitored. Initially, the weight change was analyzed based on the initial and final weights (Table 2). While no significant difference was observed in initial weights across all groups, the OVX group exhibited a significantly higher weight increase compared to the Sham group by the end of the experiment. Additionally, the OVX + E2 group exhibited a reduction in weight gain of 6.3 g; however, this was not statistically significant. Similarly, a decreasing trend was also observed in the OVX + SWP group; however, it was not significant.

### 3.4. Effect of Freeze-Dried Silkworm Powder on Uterus Weight in the OVX Mouse Model

**Figure 3:**
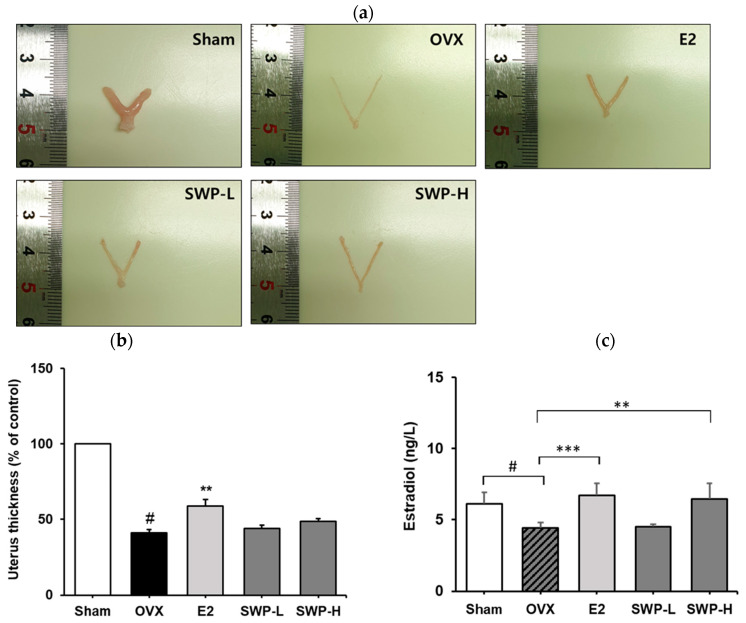
Effect of SWP on uterine thickness and serum estradiol concentration in ovariectomized (OVX) mice. OVX mice were orally administered the SWP (80 mg/kg or 160 mg/kg) or 17β-estradiol (1 mg/kg) three times a week for eight weeks. (**a**) Representative images of the uterine tissue. (**b**) Quantification of uterine thickness. (**c**) Blood 17β-estradiol levels in mice. Statistical analysis was performed using one-way ANOVA, followed by Tukey’s post hoc test using Prism 5. Data are presented as the mean ± SD (*n* = 5); ^#^ *p* < 0.001 compared to the Sham group and ** *p* < 0.01 compared to the OVX group. (**c**) Serum concentration of estradiol (pg/mL) measured using ELISA kit; ^#^ *p* < 0.001 vs. Sham group, ** *p* < 0.01 and *** *p* < 0.001 vs. OVX group.

Generally, uterine tissue atrophies, and its weight decreases as menopause progresses [31]. The reduction in uterine weight is generally attributed to the cessation of 17β-estradiol secretion, which is essential for maintaining uterine tissue [32]. Following the administration of 17β-estradiol, or SWP, the uterine tissues of mice were collected and imaged to measure their thickness (Figure 3a,b). The results showed that the uterine thickness was significantly lower in the OVX group than in the Sham group. Notably, in the E2 group, in which OVX mice received 17β-estradiol, a significant increase in uterine thickness was observed relative to the OVX group. Although the groups administered 80 mg/kg (SWP-L) and 160 mg/kg (SWP-H) of SWP exhibited an increasing trend in uterine thickness compared to the vehicle-only OVX group, these changes were not statistically significant (Figure 3b). Typically, the concentration of estradiol, an estrogen produced by the ovaries, decreases during menopause. In this study, serum estradiol levels in OVX mice were significantly lower compared to those in the Sham group, as evidenced by using an estradiol ELISA kit (Figure 3c). Conversely, in the 17β-estradiol-administered E2 group, serum estradiol levels were significantly elevated compared to those in the OVX group. In the experimental groups that received 160 mg/kg (SWP-H) of SWP, there was a trend toward increased serum estradiol levels, with statistical significance observed in the high-dose group (Figure 3c). The normalization of reduced estradiol (E2) levels in ovariectomized (OVX) animals through oral administration of silkworm pupa (SWP) at a dose of 160 mg/kg can be attributed to the presence of physiologically active components in SWP, such as phytochemicals like 1-Deoxynojirimycin and Astragalin. These compounds may play a role in regulating estrogen levels. However, to gain a more comprehensive understanding of the mechanisms underlying the improvement in blood estrogen levels by SWP, further experimental studies are necessary.

### 3.5. Effect of Freeze-Dried SWP on the Expression of Estrogen Receptor Alpha (ERα) and mRNA in Uterine Tissues of OVX Mice

**Figure 4:**
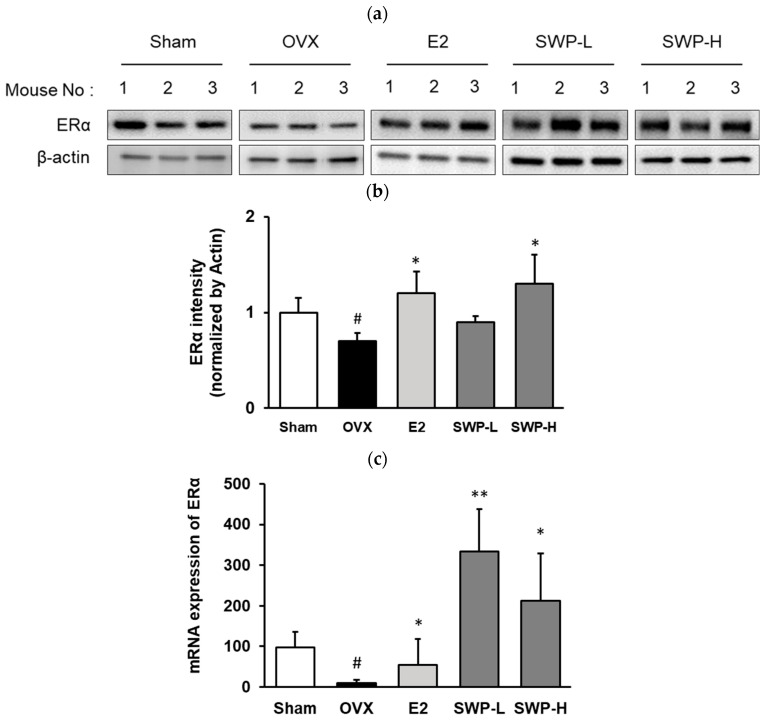
Effect of SWP on the expression of ERα in uterine tissues of ovariectomized (OVX) mice. OVX mice were orally administered the SWP (80 mg/kg or 160 mg/kg) or 17β-estradiol (1 mg/kg) three times a week for eight weeks. The uterine tissue was analyzed by immunoblotting. (**a**) Phosphorylation of estrogen receptor α in uterus tissues of ovariectomized mice. (**b**) p-ERα protein expression levels and total protein were verified with ImageJ software (version Java 8); ERα was used as a control. (**c**) mRNA expression of ERα was measured by qRT-PCR. Statistical analysis was performed using one-way ANOVA, followed by Tukey’s post hoc test using Prism 5. Data are presented as the mean ± SD (*n* = 5); ^#^ *p* < 0.05 compared to the Sham group and * *p* < 0.05, ** *p* < 0.01 compared to the OVX group.

The estrogen receptor is activated upon interaction with estrogen, which subsequently affects downstream protein and gene expression. Once treatment was completed, we analyzed the phosphorylation and gene expression of ERα in the uterine tissues of the mice. ERα expression was significantly decreased in the OVX group compared to the Sham group. Conversely, ERα was significantly elevated in OVX mice that received 17β-estradiol (E2). When comparing the OVX group to mice administered 80 mg/kg (SWP-L) and 160 mg/kg (SWP-H) of SWP, an increase in ERα expression was observed. Although this increase was not dose-dependent, it was statistically significant (Figure 4a,b). Additionally, ERα gene expression levels in uterine tissues exhibited a downward trend in the OVX group compared to the Sham group. Although both the positive control (E2) and SWP-treated groups demonstrated an upward trend in gene expression, interindividual variation prevented statistical significance (Figure 4c). Consequently, we hypothesized that the oral administration of SWP in an artificially estrogen-deprived menopausal animal model could potentially activate the estrogen receptor signaling pathway.

### 3.6. Effect of Freeze-Dried SWP on the Phosphorylation of AKT and ERK in the Uterine Tissues of OVX Mice

**Figure 5:**
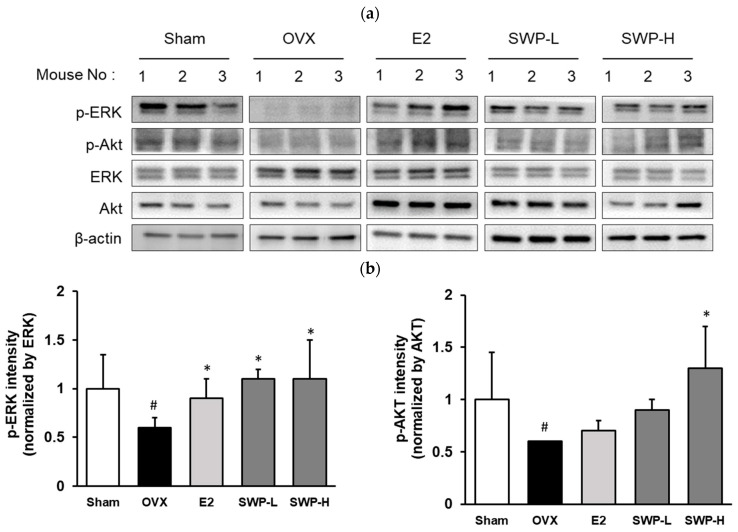
Effect of SWP on the phosphorylation of AKT and ERK in the uterine tissues of ovariectomized (OVX) mice. OVX mice were orally administered the SWP (80 mg/kg or 160 mg/kg) or 17β-estradiol (1 mg/kg) three times a week for eight weeks. AKT, ERK, and β-actin were determined by immunoblotting. (**a**) Phosphorylation of AKT and ERK in uterine tissues of OVX mice. (**b**) AKT, ERK protein expression levels, and total protein were verified with Image J software. p-AKT and p-ERK protein expression levels and total protein were verified using Image J software. Statistical analysis was performed using one-way ANOVA, followed by Tukey’s post hoc test using Prism 5. Data are presented as the mean ± SD (*n* = 5); ^#^ *p* < 0.05 compared to the Sham group and * *p* < 0.05 compared to the OVX group.

AKT and ERK are critical proteins involved in the estrogen receptor signaling pathway and are responsible for mediating signal transduction from cell membrane receptors to nuclear DNA. We compared the levels of AKT phosphorylation in uterine tissues across all treatment groups. In the OVX group, AKT phosphorylation was substantially suppressed relative to the Sham group (Figure 5). However, this suppression was significantly alleviated in the 17β-estradiol-administered positive control group. Moreover, AKT phosphorylation was significantly enhanced in the SWP-treated groups. Notably, the group that received a low dosage of SWP (SWP-L, 80 mg/kg) exhibited more robust AKT phosphorylation than the positive control group. This finding correlated with the observation for ERα phosphorylation, indicating that SWP administration could activate the estrogen receptor, thereby inducing AKT phosphorylation. Furthermore, we analyzed ERK phosphorylation, a downstream effector regulated by AKT (Figure 5). While ERK phosphorylation was diminished in the OVX group, it increased to levels comparable to those in the Sham group following the administration of the positive control (E2). A similar enhancement in ERK phosphorylation was observed in the SWP-treated groups. These findings suggest that SWP administration in the OVX mouse model not only activated the estrogen receptor but also stimulated the phosphorylation pathways of AKT and ERK.

### 3.7. Effect of Freeze-Dried SWP on Serum Levels in OVX Mice

Estrogen deficiency in menopausal women leads to metabolic syndrome and cardiovascular diseases by disrupting the balance of glucose and lipid metabolism. This imbalance results in altered body fat distribution and increased abdominal and visceral fat. Studies on ovariectomized mice showed that consuming estrogen-like substances reduces visceral fat. Estrogen’s role in regulating lipid metabolism is crucial, as its deficiency elevates triglyceride and LDL cholesterol levels, key biomarkers of cardiovascular disease. In this study, we analyzed the levels of TG, total cholesterol (TC), LDL, and HDL in the serum of mice (Table 3). The results showed that serum TG significantly increased in OVX mice compared to the Sham group. In the positive control group (OVX-E2), the increased TG levels significantly decreased, and the silkworm powder (SWP-Low, SWP-High) administration yielded a decreasing trend in triglyceride content. TC and LDL analyses showed increased levels in the OVX group, but the levels were significantly decreased in the E2-treated group. However, while TC and LDL levels tended to decrease in the SWP-administered groups, these changes were not statistically significant. However, in the low-dose silkworm powder group (SWP-L, 80 mg/kg), HDL-cholesterol significantly increased compared to the OVX group.

HDL decreased while triglycerides, TC, and LDL increased in the context of abdominal obesity [33]. This suggested that oral administration of freeze-dried silkworm powder positively impacted lipid improvement in the bloodstream. Over the eight-week administration period, liver toxicity was evaluated by analyzing serum ALT and AST levels; no significant liver damage was observed across all groups, indicating no hepatotoxicity from the SWP administration. Overall, serum analysis in the mice indicated that the administration of freeze-dried silkworm powder exerted a beneficial effect on lipid metabolism imbalance occurring in states of decreased estrogen secretion, which may help improve cardiovascular diseases.

## 4. Conclusions

Menopausal disorders include an array of physiological changes that most women experience after menopause. Currently, various pharmacological therapies are employed to improve and treat these disorders, with HRT being the predominant approach. However, HRT poses risks, including an increased incidence of endometrial cancer, heart disease, and breast cancer due to excessive estrogen levels. Consequently, it is essential to develop management strategies that can alleviate menopausal symptoms and prevent chronic diseases. Developing therapies that can mitigate the side effects of synthetic hormone treatments and prevent menopausal disorders is equally crucial. A common therapeutic approach involves consuming natural resources rich in phytoestrogens, which can act in place of estrogen. Consequently, ongoing research has focused on improving menopausal symptoms using natural substances or their combinations [34]. To evaluate the efficacy of SWP on menopausal symptoms, we administered SWP to OVX mice at doses of 80 and 160 mg/kg/day, three times a week for eight weeks, with 17β-estradiol (1 mg/mL) serving as a positive control. The SWP-L- and SWP-H-treated groups exhibited an increase in uterine thickness compared to the OVX group, which only received 0.5% MC, thus revealing that SWP affected epithelial cell thickness in the uterine walls of the OVX mice (Figure 3a,b). Biochemical assays of serum neutral lipids, specifically triglycerides, TC, HDL, and LDL cholesterol, revealed significant reductions in triglycerides in the SWP-treated groups compared to the OVX group. Furthermore, trends indicating reduced TC and LDL-cholesterol levels were also observed in the SWP groups (Table 3). These findings suggest that SWP positively impacted serum lipid profiles. Additionally, serum estradiol levels, which were significantly lower in the OVX group compared to the Sham group, increased significantly following SWP administration (Figure 3c). The phosphorylation and gene expression of ERα in the uterus were analyzed, revealing that ERα phosphorylation was significantly lower in the OVX group compared to that in the Sham group. In contrast, ERα phosphorylation in both the low- and high-dose SWP groups was significantly increased, albeit not in a dose-dependent manner (Figure 4a,b). Furthermore, significant increases in the phosphorylation of ERK and AKT, which are key in estrogen receptor signaling, suggested that SWP mediated the activation of estrogen receptor signaling (Figure 5). These results indicated that SWP can activate the estrogen receptor, increase estrogen receptor expression, and activate the relevant intracellular signaling pathways, thereby potentially alleviating various symptoms associated with decreased estrogen during menopause. Moreover, utilizing silkworms can support the agricultural industry in the Republic of Korea and serve as a substitute for imported functional foods and pharmaceuticals, thereby having a positive impact on the industry.

## Figures and Tables

**Table 1:** List of antibodies used in immunoblot analysis.

Antibodies	Source	Catalog Number	Dilution
Estrogen Receptor (ERα)	Cell Signaling Technology	#8644	1/3000
ERK	Cell Signaling Technology	#9102	1/5000
phospho-ERK	Cell Signaling Technology	#9101	1/3000
AKT	Cell Signaling Technology	#9272	1/5000
Phospho-AKT	Cell Signaling Technology	#4058	1/3000
β-Actin	Cell Signaling Technology	#4967	1/5000

**Table 2:** Effect of SWP on the body weight in OVX mice.

	Initial Weight (g)	Final Weight (g)	Body Weight Gain (g)
Sham	17.3 ± 0.56	23.1 ± 1.46	5.86
OVX	20.8 ± 0.46	28.4 ± 1.14	7.66 ^#^
Estradiol	20.6 ± 0.5	26.9 ± 2.3	6.3
SWP-Low	21.4 ± 0.45	28.9 ± 1.84	7.52
SWP-High	22.4 ± 0.61	29.86 ± 1.04	7.48

Data are presented as the mean ± standard deviation (SD) of three independent experiments. ^#^ *p* < 0.05 indicates a significant difference compared to the Sham group.

**Table 3:** Effect of SWP on the serum biochemical tests in female OVX mice.

	Sham	OVX	E2	SWP-L	SWP-H
TG (mg/dL)	20.0 ± 2.6	34.3 ± 0.6 ^#^	15.3 ± 0.6 ***	32.0 ± 1.0	29.0 ± 3.0
TC (mg/dL)	65.7 ± 0.6	94.7 ± 10.0 ^#^	71.0 ± 1.7 **	88.7 ± 2.3	85.7 ± 1.2 *
HDL (mg/dL)	38.5 ± 0.5	45.0 ± 2.9 ^#^	43.4 ± 2.6	50.6 ± 1.5 *	44.4 ± 0.6
LDL (mg/dL)	5.8 ± 0.7	7.5 ± 0.8 ^#^	4.4 ± 0.4 **	6.5 ± 0.5	6.6 ± 0.1

Data are presented as the mean ± standard deviation (SD) of three independent experiments. TG: Triglyceride, TC: total cholesterol, HDL: High-density lipoprotein cholesterol, LDL: Low-density lipoprotein cholesterol. ^#^ *p* < 0.01 vs. Sham group; * *p* < 0.05 and ** *p* < 0.01 and *** *p* < 0.001 vs. OVX group.

## Data Availability

All data is included in the article.
